# Pathophysiology of vascular ageing and the effect of novel cardio‐renal protective medications in preventing progression of chronic kidney disease in people living with diabetes

**DOI:** 10.1111/dme.15464

**Published:** 2024-11-05

**Authors:** Nikolaos Fountoulakis, Yoshihisa Miyamoto, Meda E. Pavkov, Janaka Karalliedde, Giuseppe Maltese

**Affiliations:** ^1^ School of Cardiovascular, Metabolic Medicine and Sciences King's College London London UK; ^2^ Centers for Disease Control and Prevention Atlanta Georgia USA

**Keywords:** Ageing, Diabetes, GLP‐1 receptor agonists, Diabetic Kidney Disease, SGLT‐2 inhibitors

## Abstract

**Aim:**

Among people with diabetes those with chronic kidney disease (CKD) have a reduced life expectancy with increased risk of cardiovascular disease (CVD) a major contributor to morbidity and mortality. CKD related to diabetes is growing worldwide and is one of the leading causes of kidney failure globally. Diabetes is associated with accelerated vascular ageing and the related mechanisms and mediators that drive the progression of CKD and CVD disease in people with diabetes may help provide insights into the pathophysiology of cardio‐renal complications and guide treatment interventions in people with diabetes.

**Methods:**

We conducted a narrative review of the literature using PubMed for English language articles that contained keywords that related to diabetes, chronic or diabetic kidney disease, ageing, cellular senescence, arterial stiffness, Klotho and sirtuins, sodium‐glucose co‐transporter‐2 (SGLT‐2) inhibitors, renin angiotensin aldosterone system (RAAS) and glucagon‐like peptide‐1 (GLP‐1) receptor agonists.

**Results:**

Progressive kidney disease in diabetes is associated with accelerated ageing driven in part by multiple processes such as cellular senescence, inflammation, oxidative stress and circulating uremic toxins. This accelerated ageing phenotype contributes to increased arterial stiffness, endothelial dysfunction, cognitive decline and muscle wasting, thereby elevating morbidity and mortality in individuals with diabetes and CKD. Deficiency of the kidney‐derived anti‐ageing hormone Klotho and reduced sirtuin levels play pivotal roles in these ageing pathways. Dietary, lifestyle and pharmacological interventions targeting vascular ageing may help reduce the progression of CKD and associated CVD in people with diabetes. The current standard of care and pillars of treatment for kidney disease such as RAAS inhibitors, SGLT‐2 inhibitors and GLP‐1 receptor agonists all influence pathways involved in vascular ageing.

**Conclusions:**

A multifactorial intervention to prevent the development of CKD by targeting traditional risk factors as well as treatment with novel agents with cardio‐renal beneficial effects can prevent accelerated ageing and extend lifespan in people with diabetes.

**What's new?**

- Emerging research highlights how diabetes‐related CKD is linked with accelerated vascular ageing driven by mechanisms like cellular senescence, inflammation, and oxidative stress, heightening the risk of cardiovascular disease and mortality.
- Deficiency in the kidney‐derived hormone Klotho and reduced sirtuin levels are now recognized as key drivers of ageing pathways in diabetic CKD, contributing to increased arterial stiffness and organ dysfunction.
- Novel treatments, including SGLT‐2 inhibitors, GLP‐1 receptor agonists and non‐steroidal mineralocorticoid receptor antagonists show promise not only in slowing CKD progression but also in targeting vascular ageing, offering a new strategy to reduce cardiovascular complications in people with diabetes.

## 1 INTRODUCTION

The global prevalence of diabetes has reached alarming proportions, with projections indicating a significant rise in the coming decades. Diabetes not only predisposes individuals to cardiovascular disease (CVD) but also stands as the primary cause of end‐stage kidney disease (ESKD). Despite advances in management, diabetes remains a critical factor affecting life expectancy, particularly when complicated by chronic kidney disease (CKD).^1^ Epidemiological studies reveal a reduced life expectancy among those with diabetes compared to the general population, exacerbated further by the presence of CKD.^2^ Indeed recent data from cohorts in North America and Finland of people with type 1 diabetes suggest that in the absence of kidney disease people with type 1 diabetes have a similar life expectancy compared to the general population.^3^, ^4^

In recent decades, the importance of accelerated vascular ageing and its implications in the mechanisms that drive CKD and CVD in diabetes has been demonstrated.^5^, ^6^

In light of this growing interest, we conducted a narrative review of the literature using PubMed for English language articles that contained keywords that related to diabetes, chronic or diabetic kidney disease, ageing, cellular senescence, arterial stiffness, Klotho and sirtuins, sodium‐glucose co‐transporter‐2 (SGLT‐2) inhibitors and glucagon‐like peptide‐1 (GLP‐1) receptor agonists.

In this narrative review, we will discuss how diabetes and CKD can accelerate ageing with its accompanying increased morbidity and mortality, and we will elucidate how a multifactorial intervention to improve glycemic control and tackle comorbidities in diabetes can help improve lifespan in this population. We will also describe and discuss how novel cardio‐renal protective agents in CKD may promote longevity beyond glycemic control through their pleotropic, anti‐ageing properties.

## 2 MECHANISMS OF AGEING—EFFECTS OF DIABETES ON AGEING

Ageing is characterised by the progressive deterioration of physiological functions essential for survival and fertility due to the progressive loss of tissue and organ function over time. Cellular senescence has been identified as the key driver of ageing and age‐related diseases.^7^ Age‐related diseases are conditions that are seen at an increased frequency with advancing ageing as a consequence of senescence as not all humans experience all of these pathological states although they all age.^8^ In normal—healthy—ageing organ dysfunction occurs in all individuals, and it can be viewed as the result of the normal ‘wear and tear’ over time. This chronic senescence results from long‐term macromolecular damage due to various stressors resulting in genomic instability, protein misfolding,^9^ telomere shortening,^10^ epigenetic changes,^11^ protein aggregation and dysfunction of the nuclear lamina.^12^ Age‐related diseases occur when additional stressors are imposed on a vulnerable tissue rich in senescent cells.^13^

Ageing is the accumulation of deficits that happen in every individual with a variability in regard to the rate of accumulation and the organs that are being affected. An ageing organism is characterized by a decrease in physiological reserves that make it vulnerable to extrinsic insults.

Cellular senescence is a dynamic process in which cells undergo an initial phase of transient senescence which is followed by a stable growth arrest leading to complete and late/deep senescence where there is phenotypic diversification.^14^ It is not a cell‐intrinsic process. Senescent cells produce a combination of cytokines, chemokines, extracellular matrix proteases, growth factors and other signalling molecules collectively termed senescence‐associated secretory phenotype (SASP).^15^ These signalling molecules exert pleotropic effects on neighbouring cells contributing to ageing and age‐related pathologies mainly via low‐grade chronic inflammation.^16^ Common age‐related diseases include arteriosclerosis, cancer, dementia and osteoporosis.

Diabetes can be viewed both as a result and as a promoter of cellular senescence—an age‐related disease as well as a condition that can cause accelerated systemic ageing.^17^

Pancreatic β‐cell senescence has been demonstrated to play a role in the pathogenesis of type 1, type 2 and monogenic diabetes.^18^ Hyperglycaemia‐induced superoxide activates poly (ADP‐ribose) polymerase (PARP) which leads to accumulation of polymers of ADP‐ribose that in turn inhibits the key glycolytic enzyme glyceraldehyde‐3 phosphate dehydrogenase (GAPDH).^19^ This critical step has been shown to induce the four major pathways of hyperglycaemic‐mediated tissue damage: (a) increased flux through the polyol pathway, (b) intracellular production of advanced glycation end products and (c) activation of protein kinase C (PKC) and increased flux through the hexosamine pathway.^20^ Telomere shortening has been shown in several populations in people with type 2 diabetes, a finding that has been attributed to the targeting of telomeric DNA by ROS.^21^ High glucose enhances the cell's susceptibility to oxidative DNA damage thus promoting genomic instability.^22^ Additionally, hypoglycemia—a frequent consequence of intensive glucose‐lowering treatment – has been shown to induce oxidative stress and a proinflammatory phenotype, contributing to endothelial dysfunction in both type 1^23^ and type 2 diabetes.^24^ Increased blood pressure and blood glucose levels, even within normal limits, predict a faster rate of increase of pulse wave velocity—a measure of arterial stiffness reflecting vascular ageing – compared to either one or no abnormality.^25^ Aortic pulse wave velocity (Ao‐PWV) is an independent predictor of mortality in type 2 diabetes probably reflecting an integrated, long‐term index of vascular function.^26^ Increased Ao‐PWV leads to greater transmission of pressure changes to the renal and cerebral microcirculation with the resultant exposure of these vascular beds to damaging pulsatile pressures and might serve as a link between glycaemia and accelerated kidney and cognitive function decline.^27^

## 3 CHRONIC KIDNEY DISEASE IN DIABETES AS A MODEL OF ACCELERATED AGEING

Many studies have investigated the mechanisms of kidney damage in diabetes, and the literature suggests a complex interaction of metabolic and haemodynamic factors and pathogenetic mechanisms that drive the onset and progression of CKD.^28^, ^29^

In the chronic hyperglycaemic state the non‐enzymatic glycation of proteins produces advanced glycation end‐products (AGEs)^17^ that induce podocyte damage and mesangial cell apoptosis. Activation of receptors for AGEs (RAGE)^30^ in the kidneys induce various intracellular signalling pathways such as phosphatidylinositol 3 kinase/protein kinase B (PI3K/Akt), mitogen‐activated protein kinase/extracellular regulated protein kinases (MAPK/ERK) and nuclear factor kappa‐B (NF‐κB) resulting in oxidative and endoplasmic reticulum (ER) stress as well as to inflammatory responses.^29^ Renin angiotensin aldosterone system (RAAS) activation is observed in the context of diabetes and can induce kidney damage via oxidative stress or by downregulating anti‐ageing proteins such as Sirtuins and Klotho.^31^ AGEs can also activate RAAS by stimulating angiotensinogen production in renal proximal tubular cells.^32^

Several studies have shown that in the context of the diabetic milieu kidney cells adopt a senescent phenotype. For example, in tubular epithelial cells in vitro RAGE activation results in a p21^Cip1^ mediated premature senescence as a result of ER stress.^33^ There is also a positive correlation between glomerular p16^INK4^ levels and proteinuria.^34^ A cardinal feature of senescent cells, the senescence associated secretory phenotype, includes diverse cytokines, chemokines, growth factors, proteases, and lipids that promote inflammation.^35^ SASP contributes to ECM accumulation and renal interstitial fibrosis in CKD.^36^ Chronic kidney disease can promote cellular senescence in several ways. AGEs accumulate in CKD due to increased production and reduced clearance.^37^ Interaction with RAGE activates the NF‐kB pathway,^30^ induces ER stress,^33^ downregulates autophagy and increases p16^38^ and p21 expression. Overproduction of ROS is another feature of CKD, present from the early stages of the disease as a result of the upregulation of RAAS and antioxidant enzyme deficiency. Decreased expression of nuclear factor‐erythroid 2‐related factor 2 (NRF2) – a regulator of antioxidant enzymes – in CKD is accompanied by upregulation of pro‐inflammatory markers such as NF‐kB.^39^

In a reverse‐direction Mendelian randomisation analysis a genetically predicted lower kidney function was significantly associated with a higher degree of telomere attrition linking causally CKD with telomere shortening a hallmark of cellular senescence. In the same study the reverse was also shown i.e. genetically caused shorter telomere length is associated with CKD.^40^ Mineral perturbations in CKD with resultant hyperphosphatemia have been shown to promote a systemic ageing phenotype by downregulating the expression of SIRT1. Activation of SIRT1 in vascular smooth muscle cells (VSMCs) significantly reduced phosphate‐induced senescence and calcification.

Worsening renal function leading to advanced stages of CKD produces a clinical phenotype resembling ageing.^41^ Muscle wasting is highly prevalent in ESKD and is responsible for the reduction of physical capacity, functional independence and an increase in the number of hospitalisations and mortality rates.^42^ Muscle loss and the ensuing functional decline represent a major feature of the frailty syndrome which describes a status of increased vulnerability to external and/or endogenous stressors contributing to adverse outcomes among older adults such as higher in‐hospital and all‐cause mortality, functional impairment, risk of fall, bone fractures and cognitive decline.

In a population‐based study among 322,109 people with newly diagnosed diabetes those with a diagnosis of CKD either before or after the diagnosis of diabetes had a 24% higher risk of developing frailty compared to those without CKD. Insulin resistance, low‐grade inflammation and oxidative stress might be the underlying mechanisms for the development of sarcopenia in CKD.

In 5/6th nephrectomised mice, the cell cycle arrest markers p21 and p16^INK4a^ were upregulated in the muscles of the CKD mice compared to the sham operated cohort.

Arterial stiffness is a long‐term process of structural alterations in the viscoelastic properties of the biomaterial constituting the media of the aortic wall. These alterations include fibroelastic intimal thickening, calcification of elastic lamellae, increased extracellular matrix deposition, elastynolysis and inflammation, elevated collagen along with reduced elastic fiber content.^43^ They occur from the early stages of renal impairment and progress in parallel with renal function decline leading to arterial enlargement, wall thickening and hardening. Accumulation of calcium in the walls of the aorta and the large arteries is one of the hallmarks of ageing in humans. Ageing VSMCs in the walls of arteries are characterised by a shift from a contractile phenotype to a synthetic phenotype, impaired response to contractile or diastolic mediators secreted by endothelial cells. The uremic milieu evokes key pathways leading to VSMCs adopting a senescent synthetic osteogenic phenotype.^44^ Accumulation of uremic toxins, hyperphosphatemia, oxidative stress and inflammation promote VSMCs to undergo senescence, adopt a procalcific phenotype and drive calcification in the arterial wall. Endothelial cell dysfunction is prevalent in CKD.^45^ CKD is characterized by significantly impaired endothelium‐dependent vasodilation,^46^ microvascular rarefaction^47^ and increased circulating levels of markers of endothelial cell damage. Increased oxidative stress,^48^ low grade inflammation^49^ and circulating uremic toxins^50^ have been shown to initiate the pathophysiological pathway of endothelial cell damage in the context of kidney dysfunction.

There is accumulating evidence that loss of glomerular filtration barrier and increasing albuminuria as well as reduced glomerular filtration rate are associated with accelerated decline of cognitive function.^51^ People with CKD demonstrate a faster decline in grey matter volume with higher prevalence of brain atrophy at a relatively younger age compared to age‐matched controls as shown in various imaging studies.^52^ At the molecular and cellular levels, increased oxidative and ER stress might contribute to neuronal dysfunction in CKD. Endothelial activation in the context of kidney disease in diabetes leads to degradation of the glycocalyx (the polysaccharide layer that lines the luminal endothelial surface) causing albuminuria and increased permeability in microvascular beds in other parts of the body such as the brain. Senescence of astrocytes and microglia leads them to adopt a pro‐inflammatory phenotype. Cellular senescence of endothelial cells and pericytes causes blood–brain barrier dysfunction and consequent leakage of factors like TGF‐β thereby inducing astrocyte senescence. Uremic toxins increase oxidative stress and neuroinflammation in astrocytes and microglia accelerating cognitive decline.^53^

## 4 KLOTHO AND THE SIRTUINS AS REGULATORS OF AGEING IN CHRONIC KIDNEY DISEASE

Klotho is a nephroprotective transmembrane protein predominately expressed in the renal tubules and implicated in regulating phosphate metabolism together with fibroblast‐growth factor 23 (FGF‐23).^5^ In the Greek mythology, Klotho was one the three daughters of Zeus and was believed ‘to spin the thread of life’, thereby promoting longevity.^54^ In mice, deletion of the gene encoding Klotho leads to a phenotype resembling premature human ageing including sarcopenia, cardiac hypertrophy and fibrosis, kidney atrophy, vascular calcifications, and shortened lifespan.^54^ Conversely, *in vivo* Klotho gene expression has been shown to slow the ageing process and extend survival by up to 30%.^54^, ^55^ Klotho deficiency thus may serve not only as an early biomarker for CKD but also as a critical pathogenic factor in its development and progression, influencing extrarenal complications.^5^ Klotho deficiency in CKD could enhance renal tubular and vascular senescence induced by oxidative stress, uremic toxins and high phosphate. Klotho regulates cellular senescence by interacting with multiple pathways mainly the p53/p21 and the Wnt/β‐catenin. Some of our previous work has shown that Klotho protects against VSMCs senescence via activation of Nrf2.^56^ In addition, Klotho deficiency promotes renal fibrosis in several kidney disease models.^54^, ^55^

Studies in people with type 1 and type 2 diabetes have demonstrated that circulating Klotho levels are inversely associated with the degree of albuminuria and lower levels of Klotho can predict a faster decline of kidney function in people with type 2 diabetes.^57^, ^58^ In people with type 2 diabetes and early diabetic kidney disease (DKD), circulating Klotho levels are independently and inversely associated with Ao‐PWV, the gold standard measure of arterial stiffness.^59^

Sirtuins are nicotine adenine dinucleotide (NAD)‐dependent histone deacetylases that regulate critical signalling pathways in prokaryotes and eukaryotes.^60^ Seven mammalian homologs of yeast Sir2 have been identified named SIRT1 to SIRT7. Their anti‐ageing properties have been shown to be attributed to preservation of telomere integrity, promotion of DNA repair and enhancement of genomic stability.^61^

SIRT‐1 is the most extensively studied member of the family with anti‐inflammatory and anti‐oxidative effects mediating its anti‐ageing properties.^62^ Mineral perturbations in CKD with resultant hyperphosphatemia have been shown to promote a systemic ageing phenotype by downregulating the expression of SIRT1.^63^ Activation of SIRT1 in VSMCs significantly reduced phosphate‐induced senescence and calcification.^64^ SIRT‐6‐deficient mice exhibit an ageing phenotype with reduced life‐span, loss of subcutaneous fat, reduced bone mineral density and impaired glucose homeostasis. SIRT‐6 overexpression extends lifespan in male transgenic mice potentially by reducing IGF‐1 signalling.^65^ Klotho haplodeficient mice present with 50% lower levels of circulating Klotho accompanied by increased arterial stiffness and blood pressure. Circulating Klotho deficiency downregulated the expression of vascular SIRT1 whose activation reversed increased arterial stiffness by reducing arterial collagen deposition and elastic fiber degradation. Authors concluded that the beneficial effects of SIRT1 activation in the context of Klotho deficiency were mediated by upregulation of AMPKa and consequently increased eNOS activity.^66^

## 5 HOW TO IMPROVE HEALTH AND LIFESPAN IN PEOPLE WITH DIABETES AND CHRONIC KIDNEY DISEASE

In the landmark Steno‐2 study, an intensified multifactorial intervention (including lifestyle modification and optimizing diabetes, lipid and blood pressure control) addressing cardio‐renal disease risk factors in people with type 2 diabetes and early stage CKD (microalbuminuria) significantly reduced death from any cause and from cardiovascular causes compared to conventional therapy.^67^ The mean duration of the intervention was 7.8 years after which all people were offered intensified therapy over a total follow‐up of up to 21 years. Progression to macroalbuminuria was significantly reduced in the intervention group.^68^

In a cross‐sectional study including 329 people with type 2 diabetes, those who consumed snacks and red meat were at an increased risk of being classified as having CKD compared to those with a healthier eating pattern.^69^ Adherence to a Mediterranean diet reduced the incidence of kidney disease by almost 60% in 22,187 people with type 1 and type 2 diabetes.^70^ The effect of the Mediterranean diet pattern was equally impressive in all other macro‐ and microvascular complications of diabetes.

In a large, population‐based trial people with type 2 diabetes treated with metformin had a 15% longer survival time compared to those without diabetes and 38% longer survival time compared to those taking sulphonylurea as monotherapy. Interestingly, improved survival in people on metformin compared to people without diabetes who served as controls increased with increasing morbidity.^71^ It has been shown *in vitro* that metformin may halt cellular senescence by upregulating the AMPK pathway and inhibiting the mTOR pathway via disruption of the electron transport chain of mitochondrial complex 1^72^ although ‘suprapharmacological’ concentrations required to achieve this effect cast doubt on the extrapolation of this protective action in humans.

## 6 SODIUM GLUCOSE CO‐TRANSPORTER‐2 (SGLT‐2) INHIBITORS AND AGEING IN CKD

Sodium glucose co‐transporter‐2 (SGLT‐2) inhibitors were initially used to lower blood sugar levels by promoting renal glucose excretion.^73^ Over the last few years, these agents have shown to exert cardiovascular and renal protective effects irrespective of their blood glucose‐lowering potential.^74^ The first randomised trial that showed cardiovascular benefits of SGLT2i—empagliflozin in particular—was the EMPA‐REG outcome trial where the addition of empagliflozin to standard of care in high‐risk patients reduced CV endpoints, CV death and all‐cause mortality compared to placebo.^75^

In EMPA‐REG, further analyses suggest that the reduction in CV and all‐cause mortality with empagliflozin can lead to 4.5 years of additional life expectancy at age 45 years.^76^ SGLT‐2 inhibitors have been shown to slow progression of kidney disease in both people with and without diabetes mellitus.^74^

With regard to kidney disease in people with diabetes the Canagliflozin and Kidney Events in Diabetes with Established Nephropathy Clinical Evaluation (CREDENCE) was the first study of an SGLT‐2 inhibitor to have kidney outcomes in its primary composite endpoint.^77^ People with type 2 diabetes and albuminuric CKD were randomised to receive canagliflozin 100 mg once daily or placebo. All participants had an eGFR of 30 to <90 mL/min/1.73m^2^, albuminuria [urine albumin: creatinine ratio (ACR) >33.9–565 mg/mmol (>300 to 5000 mg/g)] and received RAS blockade. Sixty per cent of recruits had an eGFR of 30–60 mL/min/1.73m^2^. The primary endpoint was a composite of ESKD (dialysis, transplantation or sustained eGFR of <15 mL/min/1.73m^2^), a doubling of the serum creatinine or death from kidney or cardiovascular causes.

The relative risk of the primary endpoint was significantly lower in the canagliflozin group with event rates of 43.2 versus 61.2 per 1000 patient‐years (HR 0.70; 95% CI 0.59–0.82; *p* = 0.00001). The relative risk of the kidney‐specific composite of ESKD, doubling of the creatinine level, or death from kidney causes was lower by 34% (HR 0.66; 95% CI 0.53–0.81; *p* < 0.001) and ESKD was lower by 32% (HR 0.68; 95% CI 0.54–0.86; *p* = 0.002).^77^ Of note, in this high‐risk population, there were no significant increases in rates of lower limb amputation or fracture.

The Dapagliflozin and Prevention of Adverse Outcomes in Chronic Kidney Disease (DAPA‐CKD) trial assessed the effect of dapagliflozin on kidney and cardiovascular events in people with CKD (both with and without type 2 diabetes).^78^ In this study, 4094 participants with an eGFR between 25 and 75 mL/min/1.73m^2^ and urine ACR of 22.6–565 mg/mmol (200–5000 mg/g) were randomised to receive dapagliflozin 10 mg once daily or placebo. The primary outcome was a composite of sustained decline in eGFR of at least 50%, ESKD, or death from kidney or cardiovascular causes. Over a median of 2.4 years, the primary outcome event occurred in 197 of 2152 participants (9.2%) in the dapagliflozin group and 312 of 2152 participants (14.5%) in the placebo group (HR 0.61; 95% CI 0.51 to 0.72; *p* < 0.001). The hazard ratio for the kidney composite of a sustained decline in eGFR of at least 50%, ESKD, or death from kidney causes was 0.56 (95% CI 0.45 to 0.68; *p* < 0.001). The effects were similar in people with type 2 diabetes to those without.^78^

The EMPA‐KIDNEY (The Study of Heart and Kidney Protection With Empagliflozin) trial included 6609 people with CKD with an eGFR 20–45 mL/min/1.73m^2^ or 45–90 mL/min/1.73m^2^ with raised urine ACR of >200 mg/g (22.6 mmol/mol) who were randomised to receive empagliflozin 10 mg once daily or placebo.^79^ The primary outcome was a composite of progression of kidney disease (defined as ESKD, a sustained decrease in eGFR to <10 mL/min/1.73m^2^ a sustained decrease in eGFR of ≥40% from baseline or death from renal causes) or death from cardiovascular causes. During a median of 2.0 years of follow‐up, progression of kidney disease or death from cardiovascular causes occurred in 432 of 3304 patients (13.1%) in the empagliflozin group and in 558 of 3305 patients (16.9%) in the placebo group (hazard ratio, 0.72; 95% confidence interval [CI], 0.64 to 0.82; *p* < 0.001).^79^

A collaborative meta‐analysis that integrated the kidney outcomes from large placebo‐controlled trials of SGLT2 inhibitors from the SGLT2 inhibitor Meta‐Analysis Cardio‐Renal Trialists' Consortium (SMART‐C) concluded that in addition to the established cardiovascular benefits of SGLT2 inhibitors, clinical evidence support their use for modifying risk of CKD progression and acute kidney injury, in patients with type 2 diabetes at high cardiovascular risk, and in patients with CKD or heart failure irrespective of diabetes status, primary kidney disease or kidney function.^80^

The SCORED (Sotagliflozin on Cardiovascular and Renal events in people with type 2 diabetes and moderate renal impairment who are at cardiovascular risk) trial recruited 10,584 people with type 2 diabetes and CKD (eGFR 25–60 mL/min/1.73m^2^). Participants were randomized in a 1:1 fashion to receive either sotagliflozin or placebo. Treatment with sotagliflozin led to a 26% reduction in the risk of the primary endpoint (HR 0.74, 95% CI 0.63 to 0.88, *p* < 0.001), which included cardiovascular death, hospitalisations for heart failure and urgent visits for heart failure compared to placebo. Unfortunately, the trial did not remain powered for endpoints examining kidney disease progression measures in the two groups because of loss of funding and early cessation, hence firm conclusions on the effect of sotagliflozin on progression of DKD cannot be excluded.^81^

The mechanisms mediating the beneficial effects of SGLT‐2 inhibitors on the cardiovascular system and the kidneys have been shown to go beyond its effect on blood glucose control.^82^ Experiments in animal models have shown that this class of drugs can delay cellular senescence by exerting its effects on several molecular pathways and have been proposed as anti‐ageing medications.^83^ SGLT‐2 inhibition mimics a state of nutrient deprivation that upregulates several pathways associated with longevity like AMPK^84^ and SIRT1^85^ which both activate FOXO3, a transcription factor that targets genes in stress resistance. Downregulation of insulin/IGF1 pathways and mTOR^86^ signalling from a reduction in glucose and circulating amino acids through the action of SGLT‐2 inhibition promote cellular repair mechanisms including autophagy and proteostasis and similarly inhibition of SGLT‐2 led to senolytic activity by indirectly upregulating the activity of AMP‐activated protein kinase.^87^

Maltese et al. suggest that the anti‐ageing effects of SGLT‐2 inhibitors may be mediated by increased expression of soluble Klotho, highlighting the need for mechanistic human studies to clarify this relationship and further investigate Klotho's role in the cardio‐renal benefits of SGLT‐2 inhibitors.

## 7 GLUCAGON‐LIKE PEPTIDE‐1 (GLP‐1) RECEPTOR AGONISTS AND AGEING IN CKD

Glucagon‐like peptide‐1 (GLP‐1) receptor agonists enhance the release of insulin from the pancreas inhibiting the secretion of glucagon from pancreatic alpha cells. Besides their role as glucose‐lowering agents they have shown substantial cardiovascular and renal protective effects. Systematic reviews and meta‐analyses suggest a clear beneficial class effect of GLP‐1 receptor agonists  on the risk of CVD and albuminuria reduction.^88^, ^89^ Currently, there is one primary kidney endpoint study reported with this class of agent in 3533 participants with T2DM and CKD where the Effects of Semaglutide on Chronic Kidney Disease in Patients with Type 2 Diabetes trial (FLOW)^90^ was evaluated. In this study, participants with T2DM with eGFR 50 to 75 mL/min/1.73 m^2^ and a urinary ACR between 33.9 mg/mmol (>300 mg/g) and 565 mg/mmol (<5000 mg/g) or an eGFR of 25 to <50 mL/min/1.73 m^2^ and ACR between 11.3 mg/mmol and 565 mg/mmol (100–5000 mg/g) were randomised to receive subcutaneous semaglutide at a dose of 1.0 mg weekly or placebo. The primary outcome was major kidney disease events, a composite of the onset of kidney failure (dialysis, transplantation or an eGFR of <15 mL/min/1.73 m^2^), at least a 50% reduction in the eGFR from baseline, or death from kidney‐related or cardiovascular causes.

This trial demonstrated both renal benefits and CVD mortality benefits with a 24% relative risk reduction of the risk of a primary outcome of major kidney disease events in the semaglutide group than in the placebo group (331 vs. 410 first events; hazard ratio, 0.76; 95% confidence interval [CI], 0.66 to 0.88; *p* = 0.0003).

GLP‐1 receptor agonists have been shown to exert beneficial effects on age‐related diseases in animal models and clinical trials. Exendin‐4 can attenuate vascular ageing by alleviating angiotensin‐II induced ROS generation in VSMCs by targeting Rac1 and Nrf2.^91^ In animal models liraglutide can significantly increase hippocampal CA1 pyramidal neuron numbers and reduce amyloid plaque formation.^92^ Long‐term treatment with dulaglutide has beneficial effects on cognitive function in people with type 2 diabetes older than 50 years of age.^93^ In an animal model of chronic stress anagliptin and exenatide ameliorated vascular endothelial senescence through upregulation of plasma adiponectin and levels of aortic PPAR‐γ, PGC‐1α and Sirt1.^94^ Human umbilical vascular endothelial cells treated with liraglutide showed increased nuclear telomerase activity mediated through the Mtorc2/Akt pathway.^95^

GLP‐1receptor agonists and DPP‐4 inhibition have been shown to prevent ageing – and diabetes related renal injury by exerting antioxidant and anti‐apoptotic effects.^73^ Indeed, in several preclinical studies GLP‐1 receptor agonists have been shown to increase Klotho levels which may in part explain the anti‐ageing effects observed with this class of medications.^96^ Similarly, several studies have also demonstrated the reduction in vascular ageing and arterial stiffness with GLP‐1 receptor agonists in people with DKD.^97^

## 8 RENIN ANGIOTENSIN ALDOSTERONE SYSTEM (RAAS) INHIBITION AND AGEING IN CKD

Overactivation of the renin angiotensin aldosterone system (RAAS) is a hallmark of CKD in diabetes.^29^ Indeed first‐line treatment for CKD in diabetes are RAAS inhibitors, which have demonstrated blood pressure independent effects on reducing progression of CKD to ESKD.^98^ The literature relating to the role of RAAS driving ageing in the kidney and cardiovascular systems has been extensively reviewed previously. Chronic activation of RAAS promotes and drives ageing associated kidney and vascular damage by several mechanisms including mitochondrial oxidative stress and SIRT. Indeed genetic studies implicate a key role for the angiotensin type 1 (AT_1_) receptor in ageing, as demonstrated by the results from animal studies which revealed that targeted disruption of the gene encoding AT_1A_ receptor can ameliorate and alleviate the ageing‐like phenotype of the heart and kidney.^99^, ^100^ In the context of Klotho, there is also evidence that the blood pressure independent effects of RAS inhibition on cardio‐renal protection in people with diabetes and CKD may be in part be driven by changes in arterial ageing as assessed by Ao‐PWV and increase in levels of Klotho.^101^, ^102^

Recent data demonstrate the benefits of finerenone, a non‐steroidal mineralocorticoid receptor antagonist (nsMRAs) on the progression of heart and kidney disease in people with type 2 diabetes and CKD.^103^ This suggests the additive and complementary role of using RAS inhibition with aldosterone antagonist to slow the progression of CKD.

Of note aldosterone has many systemic effects outside the kidney including influencing inflammation, vascular rigidity, collagen formation and driving fibrosis.^104^ The FIDELIO‐DKD trial indicated the cardio‐renal protective effects of the nsMRA finerenone, enrolling 5734 people with type 2 diabetes and CKD (eGFR ≥25 and ≤ 75 mL/min) and established on RAS inhibition with an ACE inhibitor or angiotensin type 1 receptor blocker.^105^ During a median follow‐up of 2.6 years, a primary outcome event of kidney failure, a sustained decrease of at least 40% in the eGFR from baseline or death from renal causes occurred in 504 of 2833 patients (17.8%) in the finerenone group and 600 of 2841 patients (21.1%) in the placebo group (hazard ratio, 0.82; 95% confidence interval [CI], 0.73 to 0.93; *p* = 0.001).^105^ Given the fundamental role of mineralocorticoid receptor activation in renal and cardiac fibrosis, effective and selective blocking of the signal with nsMRAs can be used in the clinical practice to prevent or slow down the progression of heart and kidney diseases as demonstrated in several past and more recent studies.^104^

### 8.1 Combination strategies of novel treatments for chronic kidney disease in diabetes

Before the introduction of SGLT2 inhibitors and GLP‐1receptor agonists, RAAS inhibitors were the mainstay of treatment for DKD for over 20 years. Landmark trials established the efficacy of SGLT2 inhibitors, GLP‐1 receptor agonists and nsMRAs in treating DKD, primarily recruiting individuals with diabetes who had or were at increased risk for CVD, most of whom were treated with RAAS blockers. However, no head‐to‐head trials or studies combining these novel agents have been conducted.

Robust evidence from post hoc analyses indicates that combining these agents may provide additive benefits in slowing kidney disease progression. For instance, a prespecified analysis of the FLOW trial (Evaluate Renal Function with Semaglutide Once Weekly) concluded that the cardiovascular and survival benefits of semaglutide occur independently of SGLT2 inhibitor treatment and may be additive.^106^ Similarly, a post hoc analyses using modelled estimates of effect, demonstrated the potential additive effects of combination therapy of SGLT2 inhibition, GLP‐1 receptor agonism, and nsMRA with RAS inhibition in a person with type 2 diabetes and albuminuria, compared to conventional care (RAS inhibition alone). This model suggested significant absolute risk reduction in CVD and progression of CKD.^103^ Indeed mechanistically the four pillars of diabetes kidney disease treatment may have complementary modes of action that would suggest potential additive benefits to address the residual risk that may be present with one or more pillars.^74^, ^107^

## 9 CONCLUSIONS

**FIGURE 1:**
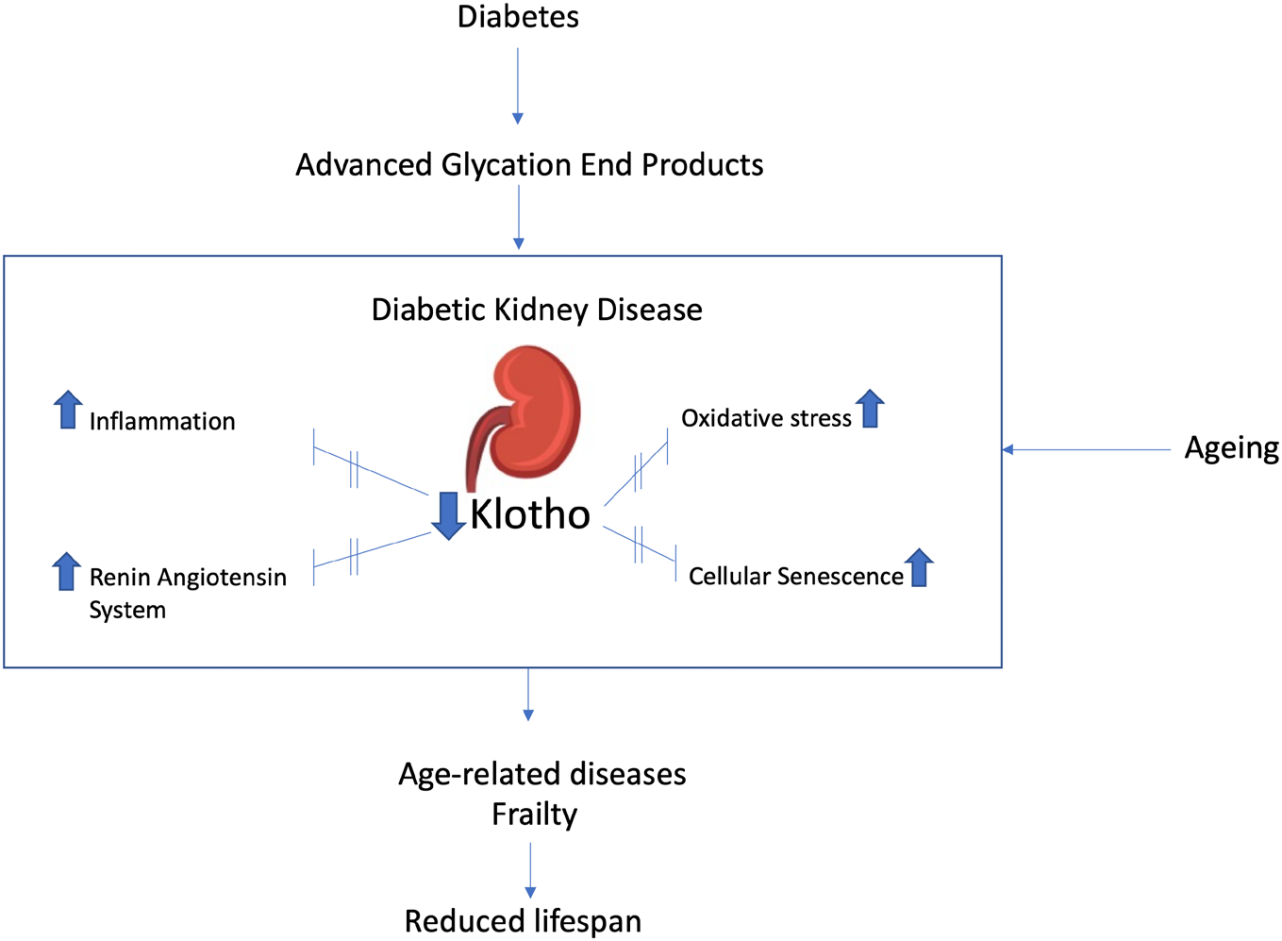
The role of Klotho in diabetic kidney disease and its implications.

Kidney damage in diabetes increases morbidity and mortality among affected individuals. Figure 1 summarizes key pathophysiological aspects of reduced lifespan in the setting of DKD. Hyperglycaemia per se can lead to a systemic ageing phenotype through multiple mechanisms such as oxidative stress and chronic low grade inflammation. This ageing process is accelerated with the onset of kidney dysfunction, even in its early stages. Vascular ageing, characterized by increased arterial stiffness and heightened pulsatile transmission of pressure waves to the microcirculation is a hallmark feature of chronic hyperglycaemia and kidney disease. The increased pulsatility to the microcirculation can exacerbate kidney damage and have a detrimental effect on both cognitive function and the coronary circulation. Diminished levels of the circulating hormone Klotho and anti‐ageing proteins from the sirtuin family, commonly observed in kidney disease, contribute to accelerated systemic ageing, as evidenced by human and animal studies.

Large epidemiological and interventional studies have shown that a healthy lifestyle along with pharmacological targeting of traditional risk factors for kidney and CVD such as hyperglycaemia, increased blood pressure and blood lipid levels can prevent the development and complications of DKD extending the lifespan of people with diabetes.

**FIGURE 2:**
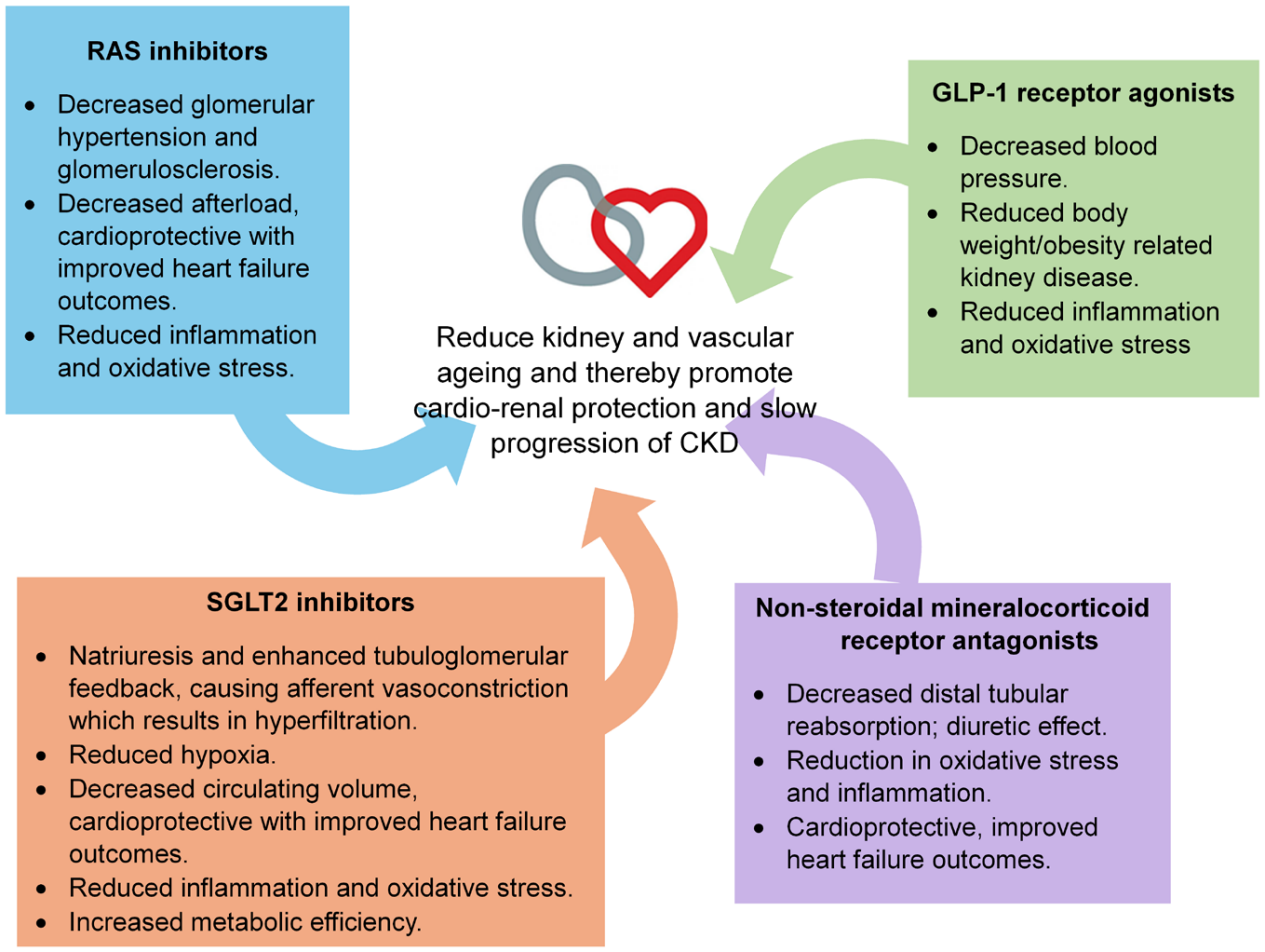
Key pillars of pharmacological treatment for chronic kidney disease. [Correction added on 9 January 2025 after first online publication: The figure 2 has been updated in this version]

In recent years, RAAS inhibition, SGLT‐2 inhibitors and GLP‐1 receptor agonists have demonstrated impressive cardiovascular and renal protective effects.^74^ As shown in Figure 2, the key pillars of pharmacological treatments for CKD and related CVD are RAAS inhibition with ACE inhibitor or angiotensin receptor blocker, SGLT‐2 inhibitors, GLP‐1 receptor agonists and nsMRAs. These pillars all have unique and complementary modes of action and beneficial effects as discussed on reducing vascular and kidney ageing via anti‐senescence mechanisms at the molecular level. These effects may in part explain their impressive results on reducing progression of CKD and associated CVD burden. Post hoc analyses of clinical trials provide compelling evidence that the combination of the aforementioned pharmacological agents has additive benefits for CVD and the progression of CKD in diabetes and set the stage for larger randomised controlled trials to establish the validity of these findings.

In conclusion, a comprehensive multifactorial intervention strategy is essential to prevent CKD onset and progression its complications. Novel therapies targeting premature ageing pathways offer promise in addressing residual cardiovascular risk and renal risk in people with diabetes and CKD.

## 10 CONFLICT OF INTEREST

The authors declare no conflict of interest.
